# LUQ view and the FAST exam: helpful or a hindrance in the adult trauma patient?

**DOI:** 10.1186/2036-7902-6-S1-A3

**Published:** 2014-01-31

**Authors:** K O’Brien, U Stolz, L Stolz, S Adhikari

**Affiliations:** 1 Department of Emergency Medicine, University of Arizona, Tucson, Arizona, USA

## Background

A rapid, accurate FAST exam is essential to the adult trauma patient evaluation. Preliminary clinical observations suggest that adequate left upper quadrant (LUQ) views are difficult for novice sonographers to obtain and rarely contribute information that cannot be gleaned from other views.

## Objective

We attempted to determine the proportion of positive FAST exams with adequate LUQ views, the proportion of these with novel information, and describe the most likely location of free fluid (FF) within the LUQ.

## Patients and methods

This was a retrospective review of ultrasound images from a QA database (Qpath) of all positive patient care FAST exams from adult trauma patients at 2 urban, academic emergency departments over 2 years. Eligible studies were reviewed for location of intra-abdominal FF. FF seen in LUQ was further characterized as in splenorenal fossa, left paracolic gutter, and/or subdiaphragmatic/suprasplenic space. Reviewers then determined the frequency resident physicians obtained LUQ images deemed adequate for medical decision making. Data are reported as proportions with 95% confidence intervals (CIs, Jeffreys method).

## Results

100 positive patient care FAST exams from adult trauma patients met eligibility criteria. While 32.0% (95% CI: 23.5-41.6%) of positive FAST exams had FF in the LUQ, only 7.0% (95% CI: 3.2-13.3%) of patients with positive FAST exam had FF isolated to LUQ. Of all patients with FF in the LUQ (n=32), 84% of patients (n=27, 95% CI: 69.1-93.8) had fluid in the paracolic gutter, with or without FF elsewhere in LUQ,.None (95% CI: 0-7.5) had fluid seen only in splenorenal fossa, 3% (n=1, 95% CI: 0.3-13.7) had FF only above spleen. 51.0% of patients (95% CI: 41.3-60.7%) had LUQ views deemed inadequate for medical decision-making.

## Conclusion

FF isolated to the LUQ occurs with a clinically relevant frequency in adult trauma patients. We propose physicians specifically obtain images of the left paracolic gutter/inferior splenic tip, as fluid in LUQ is most likely to accumulate here and is a relatively easy image to acquire for novice sonographers.
